# Respiratory Function of Fontan Pediatric Patients with Hypoplastic Left Heart Syndrome and Other Morphologies of Functionally Single Ventricle—A Pilot Study

**DOI:** 10.3390/children12050548

**Published:** 2025-04-24

**Authors:** Krzysztof Kocot, Kamil Barański, Daniel Gondko, Olga Smolarek-Kurasz, Jacek Kusa

**Affiliations:** 1 Department of Pediatric Cardiology, Faculty of Medical Sciences in Katowice, Medical University of Silesia in Katowice, 40-752 Katowice, Polandjkusa@sum.edu.pl (J.K.); 2 Department of Epidemiology, Faculty of Medical Sciences in Katowice, Medical University of Silesia in Katowice, 40-752 Katowice, Poland; kbaranski@sum.edu.pl; 3 Department of Pediatric Cardiology, John Paul II Upper Silesian Child Health Centre, 40-752 Katowice, Poland

**Keywords:** hypoplastic left heart syndrome, Fontan circulation, spirometry, fractional exhaled nitric oxide

## Abstract

**Background/Objectives**: Management of complex congenital heart defects with functionally single ventricle remains one of the greatest challenges of pediatric cardiology. The multistage surgical treatment completed with Fontan procedure is related to multiple complications. Due to non-pulsatile continuous pulmonary flow and chronic hypoxia, Fontan circulation may induce pulmonary endothelial dysfunction. However, the impact of Fontan physiology on respiratory system function is not well studied. The aim of the research was to assess respiratory function in Fontan pediatric patients with hypoplastic left heart syndrome (HLHS) and other morphologies of functionally single ventricle. The article presents the preliminary results drawn from the pilot study, focusing on Fontan patients, without a healthy children control group. **Methods**: A cross-sectional study involved Fontan patients hospitalized in the Pediatric Cardiology Clinic of the Medical University of Silesia in Katowice between August 2023 and November 2024. The exclusion criteria were lack of parental and/or patient’s consent, age < 6 years old, decompensated heart failure, asthma, atopy, respiratory infection within two weeks before the hospitalization, or significant psychomotor disability. Respiratory function assessment involved spirometry and fractional exhaled nitric oxide (FeNO) measurement. **Results**: A total of 32 patients who met inclusion criteria performed respiratory measurements. The mean age was 12.9 years old; there were 12 females. A total of 12 patients had HLHS and 20 patients had other morphologies of univentricular heart. FeNO values were relatively high with a mean of 30 ppb. Spirometry showed restrictive or mixed restrictive and obstructive ventilatory pattern. The mean forced vital capacity (FVC) levels were 79.2 ± 12.3% of predicted value (%pv) and forced expiratory volume in the first second (FEV1) 77.3 ± 13.8%pv. Children with HLHS presented statistically significantly lower percentages of predicted value of FEV1. There were statistically significant negative correlations between NT-proBNP concentrations and FEV1, FEV1%pv, MEF25-75 and MEF25-75%pv. **Conclusions**: Fontan pediatric patients present a restrictive or mixed restrictive and obstructive ventilatory pattern and relatively high FeNO levels. Patients with HLHS have worse pulmonary function than patients with other univentricular heart morphologies. This may be related to worse ventricular function in patients with HLHS.

## 1. Introduction

The management of complex cyanotic congenital heart defects (CHDs) with functionally single ventricle remains one of the greatest challenges of pediatric cardiology. In such cases, multistage surgical palliation completed with the Fontan procedure (TCPC—total cavopulmonary connection) is currently the main treatment option [1].

TCPC results in a unique circulatory system physiology, where systemic blood reaches the pulmonary vascular bed bypassing the heart. The main characteristics of such a circulation are increased systemic venous pressure, non-pulsatile, continuous pulmonary perfusion and decreased cardiac output [2]. Abnormal pattern of pulmonary flow and chronic hypoxia are factors that induce pulmonary endothelial dysfunction, resulting in increased pulmonary vascular resistance (PVR) [3]. This, together with further increase in central venous pressure, leads to Fontan circulatory failure (FCF). FCF consists of, among others, thrombotic complications, heart failure, arrhythmias, portal hypertension and liver disease, lymphatic disorders, such as protein-losing enteropathy and plastic bronchitis [2,4].

Apart from these complications, respiratory system function could be also impaired in Fontan patients. Previous studies showed frequent presence of a restrictive ventilatory pattern, which can contribute to decreased exercise tolerance and worse prognosis [5,6,7]. Due to the lack of a subpulmonary ventricle, the influence of negative intrathoracic pressure generated during inspiration on pulmonary blood flow is greater in Fontan physiology than in normal circulation [8].

The pathophysiology of negative changes in the respiratory system post TCPC is not fully understood. The most plausible theory is the negative impact of abnormal pulmonary flow pattern on lung development by causing endothelial dysfunction and remodeling [9,10]. Previous studies on respiratory function in Fontan patients concentrated mainly on spirometry and diffusing capacity of the lungs for carbon monoxide (DLCO) or nitric oxide (DLNO) [9]. Another promising and available test for functional respiratory system assessment is fractional exhaled nitric oxide (FeNO). It is considered a useful biomarker of inflammatory state in the airways and lungs and is widely used for example for the diagnosis and monitoring of asthma [11]. Nitric oxide is a crucial mediator in pulmonary endothelium and its decreased production is among the core pathophysiologic mechanisms of pulmonary hypertension (PH) [12]. Adult patients with PH were shown to have lower levels of FeNO in comparison to the healthy population [13]. The endothelial dysfunction, caused by abnormal pulmonary flow pattern in Fontan patients, might be reflected by FeNO levels, due to inflammatory state in the lungs and/or decreased nitric oxide production by the endothelium. However, the data on FeNO levels in pediatric patients with complex CHDs treated with TCPC are scarce.

Therefore, we planned a study whose aim was to assess the respiratory function of pediatric Fontan patients by performing spirometry and FeNO measurement. The additional aim was to compare the respiratory function in patients with hypoplastic left heart syndrome (HLHS) and other functionally univentricular heart morphologies. This article presents its initial results.

## 2. Materials and Methods

We conducted a single-center cross-sectional study at the Pediatric Cardiology Clinic in the Upper Silesian Child Health Center of the Medical University of Silesia in Katowice, Poland. All patients with Fontan circulation who were hospitalized in the clinic between August 2023 and November 2024 were invited to the study. The exclusion criteria were lack of parental and/or patient’s consent, age < 6 years old, decompensated heart failure, asthma, atopy, respiratory infection within two weeks before the hospitalization, or significant psychomotor disability. Atopy was defined as total immunoglobulin E (IgE) > 100 U/mL. In case of patients > 16 years old, a written consent was obtained from both the patient and his parent/guardian. Patients and parents completed the authors’ questionnaire to provide information about past medical history, treatment, symptoms and comorbidities. Additional information regarding CHD morphology and previous treatment was searched for in hospital data and patients’ medical documentation.

In patients who were eligible and agreed to the study, the blood samples for blood gas test, IgE and N-terminal prohormone of brain natriuretic peptide (NT-proBNP) assessment were taken on the second day of the hospitalization. Additionally, a saturation measurement with a pulse oximeter was performed. Then, the patients performed FeNO measurement and spirometry. The respiratory tests were conducted in the morning, to avoid the impact of circadian rhythm and before the exercise treadmill test (carried out routinely for diagnostic purposes) to avoid post-exercise changes in FeNO and spirometry. All respiratory tests were conducted by qualified personnel.

FeNO was assessed with the use of a Vivatmo pro device (Bosch, Gerlingen-Schillerhöhe, Germany). The patients performed the measurement at least twice and the arithmetic mean was calculated. We then performed spirometry with EasyOne Air (NDD, Zürich, Switzerland) in a sitting position, according to the European Respiratory Society (ERS, Lausanne, Switzerland) and American Thoracic Society (ATS, New York, NY, USA) standards. The patients repeated the maneuvers until they met ERS/ATS acceptability and repeatability criteria or the maximal number of maneuvers. Only the patients who were able to perform acceptable spirometry and/or FeNO measurement were further analyzed.

We performed statistical analysis using Statistica 13 software. (Tibco Statistica version 13.3). The categorical variables were presented as numbers and percentages, while for quantitative variables arithmetic mean, standard deviation and ranges were calculated. The spirometric parameters were presented as measured values and percentage of value predicted for age, sex, height and body mass. The normality of distribution of quantitative variables were calculated with the Shapiro–Wilk test. The comparisons between HLHS and other single ventricle morphologies were made with Student’s *t*-test for parametric variables or the Mann–Whitney U test for non-parametric variables. For comparisons of categorical variables, we used the chi square test with Yate’s correction. The correlations between NT-proBNP, a biomarker of heart failure, and respiratory parameters were assessed with Spearman’s rank correlation. The Cohen’s d was calculated by dividing the difference between means by pooled standard deviation. Alpha level 0.05 was considered as significant; however, for multiple comparisons Bonferroni’s correction was used for the variables that were the subject of investigation (FeNO, NT-proBNP and spirometric parameters).

The study protocol was approved by the Ethics Committee of the Medical University of Silesia in Katowice—agreement number BNW/NWN/0052/KB1/12/23. The funding was provided by the Medical University of Silesia in Katowice—agreement number BNW-1-033/N/3/K.

## 3. Results

During the study period, 36 patients with functionally univentricular heart and TCPC met the inclusion criteria and were qualified to the study. Of them, 32 were able to perform acceptable spirometry and/or FeNO measurement. According to the study protocol, four patients who were not able to cooperate and complete any respiratory measurement were not included in the statistical analysis. Of 32 patients included, four performed acceptable spirometry, but could not complete acceptable FeNO measurement, and three were able to complete FeNO, but not spirometry. The basic characteristics of the study group are presented in Table 1.

In the study group there were 12 patients with HLHS. When compared with other Fontan patients, they had statistically significantly lower oxygen saturations on pulse oximetry and higher NT-proBNP levels. No other statistically significant differences between the groups in terms of basic characteristics were observed. A detailed comparison of HLHS and other patients is presented in Table 2.

The average FeNO level in the study group was 30 ppb. The spirometry showed restrictive or mixed obstructive and restrictive pattern with mean forced vital capacity (FVC) and forced expiratory volume in 1 s (FEV1) below 80% of value predicted. The results of FeNO and spirometry are presented in Table 3.

There were no statistically significant differences between HLHS and other Fontan patients in FeNO levels. In terms of spirometry, patients with HLHS had statistically significantly lower percentages of predicted value of FVC, FEV1 and MEF25-75. After Bonferroni correction, the difference remained significant for FEV1. All HLHS patients had FEV1 below 80% of predicted value in comparison to 42% of other patients (*p* = 0.008). The results of FeNO and spirometry in HLHS and other Fontan patients are shown in Table 4.

The correlation analysis showed statistically significant negative correlations between NT-proBNP concentrations and FEV1, FEV1%pv, MEF25-75 and MEF25-75%pv. The results of the correlation analysis are presented in Table 5.

## 4. Discussion

Our study showed that pediatric patients with functionally univentricular heart post TCPC present decreased lung function in terms of spirometry. This was also observed in other, rather rare studies that assessed lung function in Fontan patients. Mild to significant limitations in lung volumes, especially FVC, are commonly found in this group, as shown in a recent systematic review [14,15]. The reason for this fact is not well understood. However, one of the possible explanations might be the negative impact of Fontan circulation on lung development. A study from Denmark showed that several spirometric parameters declined over a 10-year period of time in a cohort of pediatric Fontan patients. Such a change was not observed in adult patients [9]. The exact pathophysiology of impaired lung function after TCPC is not well studied. The most plausible theory is the negative impact of non-pulsatile blood flow on pulmonary vascular development [8]. Lack of pulsatile flow may induce endothelial dysfunction, expressed by reduced activity of endothelial nitric oxide synthetase (eNOS). In addition, chronic hypoxia through hypoxia-inducible factors (HIFs) activation may increase the levels of endothelin-1 and further promote pulmonary vasculature maladaptive remodeling leading to increased PVR [3]. It is hypothesized that this may alter pulmonary alveolar development and growth in childhood resulting in restrictive lung disease [8]. Apart from that, other factors related to the CHD and its treatment such as multiple operations, mechanical ventilations, pleural adhesions or phrenic nerve palsy might contribute to impairments in the pulmonary function of Fontan patients [5,8].

An important finding of our study is that the Fontan pediatric patients with HLHS, and therefore with a single right ventricle (RV), had worse pulmonary function than those with other univentricular heart morphologies. Many previous studies showed that this group of patients had a worse prognosis with greater risk of complications and mortality [16,17,18]. As observed in a large historical cohort study, a morphologic RV was negatively associated with long-term survival. The patients with a morphologic RV presented a tendency toward progressive atrioventricular valve regurgitations and ventricular dysfunction [18]. Due to its morphological structure, the RV might be more prone to failure when forced to act as a systemic ventricle. It seems plausible that, similarly to other Fontan complications, declined respiratory function would be more frequent in patients with a morphologic RV. However, other studies assessing respiratory function in Fontan patients either did not focus on single ventricle morphology or did not observe statistically significant differences [14].

In our study, the patients with HLHS had statistically significantly lower percentages of predicted values of FEV1 in comparison to other Fontan patients. This difference could not be explained by anthropometric variables, time from TCPC operation, age at TCPC or pharmacological treatment. The only factor that differed between the children with HLHS and those with other single ventricle morphologies were oxygen saturations and NT-proBNP concentrations. HLHS Fontan patients had statistically significantly lower oxygen saturations and higher NT-proBNP levels. Even though the patients qualified for the study were in clinically stable condition, higher NT-proBNP might reflect worse, perhaps subclinical, ventricular function in patients with single RV morphology. We believe that worse ventricular function and hypoxia might accelerate the negative changes in pulmonary vasculature described above, further impairing pulmonary alveolar development and growth. In line with this hypothesis, several statistically significant negative correlations between NT-proBNP levels and spirometric parameters were observed.

In contrast to spirometric parameters, there were no statistically significant differences in FeNO levels between the patients with HLHS and other patients. At the beginning of the study, we hypothesized that FeNO measurement would be useful in determining patients with worse pulmonary vasculature remodeling and potentially worse lung function. Some studies in adult patients with idiopathic pulmonary hypertension showed that FeNO levels were negatively associated with pulmonary hypertension severity [19]. A study from Japan showed that in adult CHD patients lower FeNO values were associated with cyanosis, but no associations with right heart catheterization hemodynamic parameters were observed [20]. The authors explained that low FeNO levels in cyanotic patients might reflect the endothelial dysfunction induced by hypoxia. However, the described group of patients was very heterogenic and only a few of them had single ventricle morphology. To our knowledge, the current study is the first to assess FeNO levels in single ventricle pediatric patients post TCPC.

FeNO values observed in the study group were relatively high, with a mean value of 30 ppb—intermediate values according to the American Thoracic Society [21]. In comparison, a study on randomly selected school children aged 6–9 years old from four cities in Silesia region, Poland, showed that children without respiratory symptoms with and without overweight or obesity had mean FeNO levels of 13.8 and 15.0, respectively [22]. Our own preliminary data from the control group (n = 12, the study is ongoing) showed mean values of 25 ppb. As described above, endothelial dysfunction is normally related to decreased eNOS activity and lower NO production by endothelial cells [3]. However, the data on exact pathophysiology of endothelial remodeling in Fontan patients are scarce, especially in children. In this group, the mechanisms responsible for pulmonary vascular remodeling might be different than in patients with biventricular circulation and pulmonary hypertension [3]. For example, it was shown that pulmonary vasculature remodeling in Fontan patients is characterized by intimal proliferation and medial thinning, rather than medial hyperplasia [23]. Higher than expected FeNO levels in Fontan patients might be due to increased inducible NOS, involved in inflammatory processes. This may indicate that inflammation plays an important role in impaired respiratory function in patients post TCPC. Another explanation could be that the main source of FeNO in this group is not the endothelium, but rather the airways. Elevated FeNO levels might reflect subclinical airway inflammation.

Without additional research including more advanced methods (for example inflammatory cytokines levels, white blood count or breathing air condensate analysis), the inflammatory hypothesis cannot be confirmed.

### 4.1. Limitations

The study has several limitations. Firstly, it was a pilot study and did not include a control group. This may weaken or make impossible potential comparisons with healthy children. Secondly, the cross-sectional design of the study limits the ability to draw conclusions on causality. Apart from single ventricle morphology, multiple other factors might impair TCPC patients’ lung function, for example mechanical ventilation, phrenic nerve palsy or other surgical complications. In addition, the study group is relatively small, which may influence the statistical significance of the comparisons, but also increase the risk of bias. Nevertheless, many of the cited studies, especially those on pediatric Fontan patients, included similar or fewer number of Fontan patients.

### 4.2. Future Study Directions

The initial results of the pilot study show opportunities for the future research, but also limitations that should be addressed. The authors plan to continue this research and are currently recruiting a control group of patients without CHDs admitted to the Pediatric Cardiology Clinic. The control patients are being examined in the same clinical environment with the same methods, to make comparisons more reliable. In the next step, multicenter cooperation might enable us to verify the results on larger study groups.

Finally, larger and well-designed longitudinal studies are needed in order to address limitations related to cross-sectional study design. Such prospective case-control studies might imply additional methods like breathing air condensate analysis or cytokine levels analysis to verify the inflammatory hypothesis. In addition, potential confounding factors might be better controlled in a prospective study with repeated respiratory function assessments.

This approach may enhance our knowledge of respiratory function and respiratory–circulatory interdependencies in pediatric Fontan patients.

## 5. Conclusions

Fontan pediatric patients present a restrictive or mixed restrictive and obstructive ventilatory pattern and relatively high FeNO levels. Patients with HLHS have worse pulmonary function than patients with other single ventricle heart morphologies. This may be related to worse ventricular function in patients with HLHS.

## Figures and Tables

**Table 1:** Basic characteristics of the study group (n = 32).

Variable	X	SD	Range
Age [years]	12.9	3	7–18
Height [m]	1.5	0.2	1.2–1.8
Body mass [kg]	43.7	15.1	23–76.5
BMI [kg/m^2^]	18.5	3.2	14–25.6
Age at the Fontan operation [months]	40.6	25.8	14–144
Time from the Fontan operation [months]	120.3	45	23–216
SpO_2_ [%]	89.9	5.6	76–98
SO_2_ in arterial blood [%]	89.1	5.6	71.9–96.4
NT-proBNP [pg/mL]	160.1	165.8	14.6–808.8
	n **(%)**		
Sex—female	12 (37.5)		
**Diagnosis**	n **(%)**		
HLHS	12 (37.5)		
AT	5 (15.6)		
DILV	4 (12.5)		
SV	5 (15.6)		
Other morphologies	6 (18.8)		
**Fontan pathway**	n **(%)**		
Extracardiac conduit	32 (100)		
Patent fenestration	16 (57.1)		
**Pharmacotherapy**	n **(%)**		
ASA	29 (90.6)		
VKAs	3 (9.4)		
ACE-Is	27 (84.4)		
Spironolactone	25 (78.1)		
BBs	9 (28.1)		
Sildenafil	13 (40.6)		
**Symptoms**	n **(%)**		
Decreased exercise tolerance	19 (59.4)		

Legend: n—number of observations, X—arithmetic mean, SD—standard deviation, BMI—body mass index, SpO_2_—oxygen saturation in pulse oximetry, SO_2_—oxygen saturation in blood gas test, HLHS—hypoplastic left heart syndrome, AT—tricuspid atresia, DILV—double inlet left ventricle, SV—single ventricle, ASA—acetylsalicylic acid, VKAs—vitamin K antagonists, ACE-Is—inhibitors of angiotensin-converting enzyme, BBs—beta blockers.

**Table 2:** Basic characteristics of HLHS patients (n = 12) and other Fontan patients (n = 20).

Variable	HLHS (n = 12)	Other (n = 20)	*p* *
	X ± SD	X ± SD	
Age [years]	13 ± 2.5	12.8 ± 3.4	0.8
Height [m]	1.5 ± 0.1	1.5 ± 0.2	0.9
Body mass [kg]	40.9 ± 10.4	45.5 ± 17.4	0.4
BMI [kg/m^2^]	17.6 ± 2.6	19.1 ± 3.5	0.2
Age at the Fontan operation [months]	32.7 ± 9.7	45.3 ± 31.1	0.5
Time from the Fontan operation [months]	126.6 ± 39.1	116.6 ± 48.8	0.5
SpO_2_ [%]	87.4 ± 7.1	91.4 ± 4	0.04
SO_2_ in arterial blood [%]	88.2 ± 6.9	89.6 ± 4.7	0.4
NT-proBNP [pg/mL]	227.4 ± 140.7	119.7 ± 169.7	0.004
	n **(%)**	n **(%)**	*p* **
Sex—female	3 (25)	9 (45)	0.4
**Fontan pathway**	n **(%)**	n **(%)**	*p* **
Patent fenestration	5 (55.6)	11 (57.9)	0.7
**Pharmacotherapy**	n **(%)**	n **(%)**	*p* **
ASA	11 (91.7)	18 (90)	0.6
VKAs	1 (8.3)	2 (10)	0.6
ACE-Is	10 (83.3)	17 (85)	0.7
Spironolactone	9 (75)	16 (80)	0.9
BBs	3 (25)	6 (30)	0.9
Sildenafil	7 (58.3)	6 (30)	0.2
**Symptoms**	n **(%)**	n **(%)**	*p* **
Decreased exercise tolerance	7 (58.3)	12 (60)	0.7

Legend: n—number of observations, X—arithmetic mean, SD—standard deviation, BMI—body mass index, SpO_2_—oxygen saturation in pulse oximetry, SO_2_—oxygen saturation, HLHS—hypoplastic left heart syndrome, AT—tricuspid atresia, DILV—double inlet left ventricle, SV—single ventricle, ASA—acetylsalicylic acid, VKAs—vitamin K antagonists, ACE-Is—inhibitors of angiotensin-converting enzyme, BBs—beta blockers; * Student’s *t*-test/Mann–Whitney U test; ** chi square test with Yates correction.

**Table 3:** Results of FeNO and spirometry in the study group.

Variable	X	SD	Range
FeNO [ppb]	30.0	12.1	11.5–52.3
FVC [l]	2.5	1.0	1.3–5.3
FVC%pv [%]	79.2	12.3	59–110
FEV1 [l]	2.1	0.9	1.1–4.4
FEV1%pv [%]	77.3	13.8	51–108
FEV1/FVC [%]	83.7	17.7	7.7–99.2
FEV1/FVC%pv [%]	97.4	11.6	69–114
MEF25-75 [l]	2.6	1.3	0.8–5.5
MEF25-75%pv [%]	77.8	27.7	29–122

Legend: X—arithmetic mean, SD—standard deviation, FeNO—fractional exhaled nitric oxide, FVC—forced vital capacity, FEV1—forced expiratory volume in 1 s, FEV1/FVC—Tiffeneau index, MEF25-75—mid-expiratory flow between 25% and 75% of vital capacity; %pv —percentage of value predicted.

**Table 4:** Results of FeNO and spirometry—comparison of patients with HLHS (n = 12) and other Fontan patients (n = 20).

Variable	HLHS	Other	*p* **	Effect Size
	X ± SD	X ± SD		
FeNO [ppb]	29.0 ± 12.7	30.5 ± 12.1	0.7	−0.05
FVC [l]	2.3 ± 0.7	2.6 ± 1.4	0.4	−0.24
FVC%pv [%]	72.1 ± 11.2	83 ± 11.4	0.02 ***	−0.97
FEV1 [l]	1.8 ± 0.6	2.3 ± 1.	0.2	−0.54
FEV1%pv [%]	65.7 ± 9.5	83.4 ± 11.7	<0.001	−1.60
FEV1/FVC [%]	74.2 ± 26.7	88.6 ± 7.6	0.08	−0.95
FEV1/FVC%pv [%]	92 ± 15	100.2 ± 8.6	0.06	−0.72
MEF25-75 [l]	2.2 ± 1.2	2.9 ± 1.3	0.1	0.55
MEF25-75%pv [%]	63 ± 27.9	85.6 ± 24.9	0.03 ***	−0.81

Legend: n—number of observations, X—arithmetic mean, SD—standard deviation, FeNO—fractional exhaled nitric oxide, FVC—forced vital capacity, FEV1—forced expiratory volume in 1 s, FEV1/FVC—Tiffeneau index, MEF25-75—mid-expiratory flow between 25% and 75% of vital capacity; %pv —percentage of value predicted; ** Student’s *t*-test/Mann–Whitney U test; *** not statistically significant after Bonferroni’s correction.

**Table 5:** Spearman’s rank correlation between NT-proBNP concentrations and respiratory measurements.

Variable	R	*p*
FeNO	−0.28	0.1
FVC	−0.29	0.1
FVC%pv	−0.23	0.2
FEV1	−0.43	0.02
FEV1%pv	−0.45	0.02
FEV1/FVC	−0.35	0.06
FEV1/FVC%pv	−0.31	0.1
MEF25-75	−0.42	0.02
MEF25-75%pv	−0.37	0.04

Legend: FeNO—fractional exhaled nitric oxide, FVC—forced vital capacity, FEV1—forced expiratory volume in 1 s, FEV1/FVC—Tiffeneau index, MEF25-75—mid-expiratory flow between 25% and 75% of vital capacity; R—correlation coefficient; %pv—percentage of value predicted.

## Data Availability

The dataset is available on request from the authors.

## Abbreviations

The following abbreviations are used in this manuscript:

FeNO	fractional exhaled nitric oxide
HLHS	hypoplastic left heart syndrome
AT	tricuspid atresia
DILV	double inlet left ventricle
SV	single ventricle
ASA	acetylsalicylic acid
VKA	vitamin K antagonists
ACE-I	inhibitors of angiotensin converting enzyme
BB	beta blockers
CHD	congenital heart defects
PVR	pulmonary vascular resistance
FCF	Fontan circulatory failure
DLCO	diffusing capacity of the lungs for carbon monoxide
DLNO	diffusing capacity of the lungs for nitric monoxide
IgE	immunoglobulin E
NT-proBNP	N-terminal prohormone of brain natriuretic peptide
FVC	forced vital capacity
FEV1	forced expiratory volume in 1 s
FEV1/FVC	Tiffeneau index
MEF25-75	mid-expiratory flow between 25% and 75% of vital capacity
eNOS	endothelial nitric oxide synthetase
BMI	body mass index
SpO2	oxygen saturation in pulse oximetry
SO2	oxygen saturation
TCPC	total cavopulmonary connection
